# LLPSDB v2.0: an updated database of proteins undergoing liquid–liquid phase separation *in vitro*

**DOI:** 10.1093/bioinformatics/btac026

**Published:** 2022-01-13

**Authors:** Xi Wang, Xiang Zhou, Qinglin Yan, Shaofeng Liao, Wenqin Tang, Peiyu Xu, Yangzhenyu Gao, Qian Li, Zhihui Dou, Weishan Yang, Beifang Huang, Jinhong Li, Zhuqing Zhang

**Affiliations:** College of Life Sciences, University of Chinese Academy of Sciences, Beijing 100049, China; College of Life Sciences, University of Chinese Academy of Sciences, Beijing 100049, China; College of Life Sciences, University of Chinese Academy of Sciences, Beijing 100049, China; College of Life Sciences, University of Chinese Academy of Sciences, Beijing 100049, China; College of Life Sciences, University of Chinese Academy of Sciences, Beijing 100049, China; College of Life Sciences, University of Chinese Academy of Sciences, Beijing 100049, China; College of Life Sciences, University of Chinese Academy of Sciences, Beijing 100049, China; College of Life Sciences, University of Chinese Academy of Sciences, Beijing 100049, China; College of Life Sciences, University of Chinese Academy of Sciences, Beijing 100049, China; College of Life Sciences, University of Chinese Academy of Sciences, Beijing 100049, China; College of Life Sciences, University of Chinese Academy of Sciences, Beijing 100049, China; College of Life Sciences, University of Chinese Academy of Sciences, Beijing 100049, China; College of Life Sciences, University of Chinese Academy of Sciences, Beijing 100049, China

## Abstract

**Summary:**

Emerging evidences have suggested that liquid–liquid phase separation (LLPS) of proteins plays a vital role both in a wide range of biological processes and in related diseases. Whether a protein undergoes phase separation not only is determined by the chemical and physical properties of biomolecule themselves, but also is regulated by environmental conditions such as temperature, ionic strength, pH, as well as volume excluded by other macromolecules. A web accessible database LLPSDB was developed recently by our group, in which all the proteins involved in LLPS *in vitro* as well as corresponding experimental conditions were curated comprehensively from published literatures. With the rapid increase of investigations in biomolecular LLPS and growing popularity of LLPSDB, we updated the database, and developed a new version LLPSDB v2.0. In comparison of the previously released version, more than double contents of data are curated, and a new class ‘Ambiguous system’ is added. In addition, the web interface is improved, such as that users can search the database by selecting option ‘phase separation status’ alone or combined with other options. We anticipate that this updated database will serve as a more comprehensive and helpful resource for users.

**Availability and implementation:**

LLPSDB v2.0 is freely available at: http://bio-comp.org.cn/llpsdbv2.

**Supplementary information:**

Supplementary data are available at *Bioinformatics* online.

## 1 Introduction

Biomolecules, mostly including proteins and nucleic acids can form liquid-like membraneless condensates, such as membraneless organelles (MLOs), via liquid–liquid phase separation (LLPS) (Alberti *et al.*, 2019; Dolgin, 2018). The LLPS of proteins has drawn a lot of interests recently not only because mounting evidences indicate its biological importance in life processes, but due to its relevance to couples of incurable diseases(Uversky, 2017), such as amyotrophic lateral sclerosis (ALS), frontotemporal dementia (FTD) and Alzheimer’s disease (AD) (Kostylev *et al.*, 2018; Mann *et al.*, 2019; Patel *et al.*, 2015).

The multivalent weak interactions between proteins or between proteins and RNA/DNA have been deemed as the driving forces of the formation of liquid condensates through LLPS (Li *et al.*, 2012), which arise from the properties of biomolecules themselves. Meantime, LLPS processes of proteins are also heavily influenced by a variety of environmental conditions such as temperature, pH, pressure, ionic strength and crowding agent, etc. (Zhang and Lai, 2020). As a result, thorough understanding the mechanism of LLPS as well as its biological function through proteome-scale experimental screening of proteins does not seem feasible. Comprehensive curation of experimentally confirmed LLPS proteins would provide valuable information for biomolecular LLPS investigation. Based on the mounting data generated in protein LLPS experiments or component identification of particular MLOs in recent years, several databases [LLPSDB (Li *et al.*, 2020b), PhaSePro (Meszaros *et al.*, 2020), PhaSepDB (You *et al.*, 2020), DrLLPS (Ning *et al.*, 2020), RNAgranuleDB (Youn *et al.*, 2019)] have been developed. To our knowledge, the LLPSDB (http://bio-comp.org.cn/llpsdb) is the only released database designed specifically for proteins undergoing LLPS that have been validated by experiments *in vitro* (Li *et al.*, 2020a). It incorporated both natural and designed proteins and provided the detailed information of them as well as corresponding specific experimental conditions at which they undergo LLPS *in vitro*. Since LLPSDB was released, the data have been downloaded more than 4000 times and several prediction tools of LLPS protein have been developed based on it (Hardenberg *et al.*, 2020; Raimondi *et al.*, 2021; Saar *et al.*, 2021; Sun *et al.*, 2019).

With the rapid increase in the studies of protein LLPS and the growing popularity of LLPSDB, we developed an updated version of the database—LLPSDB v2.0 by adding newly discovered systems that undergo LLPS *in vitro*. A new class ‘Ambiguous system’, which includes unclear components but still can undergo LLPS, is recorded. In total, LLPSDB v2.0 contains 2917 experimentally validated entries including 586 independent proteins. Compared with the previous version, LLPSDB v2.0 has been made a significant improvement both in data quantity and in web interface.

## 2 Materials and methods

### 2.1 Data collection and curation

All data in LLPSDB v2.0 were curated manually from published literatures and related resources. The literatures were screened via retrieving in ‘PubMed’ and ‘Web of Science’ by key words: phase separation, phase transition, liquid, protein, demixing, assembly, condensate, condensation, coacervate, segregate and segregation, same as the previous version LLPSDB. Finally, 167 research articles reported newly from July 2019 to March 2021 were collected. Totally, 321 literatures were curated in the updated version LLPSDB v2.0.

In LLPSDB v2.0, each entry is defined based on specific protein sequence and nucleic acid type (such as wild-type FUS and mutated FUS belong to different entries, similarly, component of RNA with 10 nt and that with 20 nt belong to different entries), not based on experimental conditions (such as salt concentration, buffer molecules and crowding agents, along with temperature, pressure and pH, etc.), same as LLPSDB. Those entries in which the components in the experimental systems are clear classified as ‘Unambiguous system’, and the rules of data collection, curation and management for this class are consistent with the previous version. A new classification named as ‘Ambiguous system’ is identified when an experimental system contains unclear (or unconfirmed) proteins (such as protamine, which is a mixture of naturally occurring arginine-rich peptides, see the Entry with ID: LLPSC000075), or part or all ingredients (proteins and/or RNA/DNA) are integrated as one component in the LLPS experiment (such as chromatin, nucleosome, etc.) (Gibson *et al.*, 2019; Sanulli *et al.*, 2019). In some cases, ambiguous system includes clear protein or RNA/DNA component(s). For the unclear part, we call it as ‘complex’. For example, S, Sanulli *et al.* show that chromatin compaction by the S*chizosaccharomyces pombe* HP1 protein Swi6 results in phase-separated liquid condensates (Sanulli *et al.*, 2019). In this system, the Swi6 is considered as clear protein component and the nucleosome arrays as ‘complex’ (Entry ID: LLPSC000001). For these systems, the specific experimental conditions were recorded, but annotations of the complex (unclear part) were left out only with a brief description kept. For the clear protein in this classification, annotations of wild-type were shown in a detailed protein page (same as ‘Unambiguous system’). All collected data in LLPSDB v2.0 were at least double-checked. Any incomplete/ambiguous information had been consolidated either by contacting the corresponding author(s) of the article or tracking related references.

Protein annotations (mainly for natural proteins) such as localization, Gene Ontology (GO) term and sequence (if not provided by literature) in LLPSDB v2.0 were obtained from UniProt/NCBI, while the rest of annotations were mining and curated from other related databases. The linkages of the related databases [such as DisProt (Quaglia *et al.*, 2021), OMIM (Amberger *et al.*, 2019), IDEAL (Fukuchi *et al.*, 2014), AmyPro (Varadi *et al.*, 2018), FuzDB (Hatos *et al.*, 2021), etc.] for each protein were updated with the newest versions. The intrinsically disordered regions (IDRs) and low complex regions (LCRs) extracted from MobiDB were changed slightly due to it has been updated recently (Piovesan *et al.*, 2021).We selected the ‘features ID’ annotated by MobiDB4.0—‘curated-disorder-merge’ (or ‘prediction-disorder-priority’, if the former is not provided) for IDRs and ‘prediction-low_complexity-merge’ for LCRs in LLPSDB v2.0.

### 2.2 Implementation of web services

To provide a more user-friendly interface and more stable application of web service, we improved web implementation of LLPSDB v2.0. The data were stored and organized through MySQL. HTML, Javascript and PHP were used for interface design and website background management.

## 3 Results

### 3.1 Datasets updating and statistics

LLPSDB v2.0 has been made a significant improvement in data quantity. The overall comparison of data volume between LLPSDB and LLPSDB v2.0 is shown in Table 1. In LLPSDB v2.0, total 2917 manually collected entries are included, and 586 independent proteins, as well as 6678 experimental conditions (including 448 phase diagrams with each of them counted as one experimental condition) were curated from 321 literatures published up to March 2021. Therefore, the data volume of LLPSDB v2.0 is more than twice as large as that of the previous version LLPSDB.

**Table 1.** Data volume comparison between LLPSDB and LLPSDB v2.0

Items	Number in LLPSDB	Number in LLPSDB v2.0
Entries	1175	2917
Independent proteins	273	586
Experimental conditions	2392	6678
Literatures	154	321

More specific comparison of the data subsets in LLPSDB versus LLPSDB v2.0 is shown in Table 2.

**Table 2.** Overview of the data subsets in LLPSDB versus LLPSDB v2.0

Classifications	Data types	Number in LLPSDB	Number in LLPSDB v2.0
Protein type	Natural protein	198	435
	Designed protein	75	151
Phase separation status	Phase separation	1483	4271
	No phase separation	700	1959
	Phase diagram	209	448
Main components type (Entries)	Protein(s)	908	1945
	Protein(s) + RNA	182	617
	Protein(s) + DNA	86	259
Main components number (Entries)	One component	536	1230
	Two components	501	1302
	More components	138	289
Ambiguous systems (Entries)	Protein(s) + complex	—	56
	Complex	—	40

For all independent proteins deposited in the databases, there are 435 natural occurring proteins and 151 engineering designed ones in LLPSDB v2.0, and the corresponding numbers are 198 and 75 in LLPSDB. Among the natural proteins in LLPSDB v2.0, there are 389 proteins from eukaryote (316 from animals, 15 from plants and 58 from fungus), 30 from prokaryote and 16 from virus (a new emerging group of proteins in LLPSDB v2.0) (Supplementary Table S1). Natural proteins can also be grouped according to their biological function annotations based on GO terms. A ‘GO-tag’ is defined as one group in which at least 10 collected independent proteins possess the same GO term. There are 15 GO-tag groups in LLPSDB v2.0, four more than LLPSDB—‘dimerization activity’, ‘inhibitor activity’, ‘adaptor activity’ and ‘activator activity’ (Supplementary Table S2).

For the total 6678 experimental conditions in LLPSDB v2.0, 4271 of them belong to phase separated status, 1959 of them belong to ‘no LLPS’ status and 448 of them correspond to phase diagrams. The numbers of all the three subsets are 2–3 times as large as those in the previous version LLPSDB. Although we only collected the data that proteins undergoing LLPS *in vitro*, most of the investigated systems (corresponding 3347 experimental conditions) have LLPS evidences *in vivo*/in cell at the meantime.

The components in each entry in LLPSDB v2.0 may be specific protein(s), protein(s) and RNA/DNA or obscured complex. Therefore, the entries are grouped into ‘Unambiguous system’ or ‘Ambiguous system’. In ‘Unambiguous system’ group, similar as LLPSDB, the 2917 entries can be categorized into ‘Protein(s)’ (1945), ‘Protein(s) + RNA’ (617) and ‘Protein(s) + DNA’ (259) according to specific components, or can be categorized into ‘One component’ (1230), ‘Two components’ (1302) and ‘more components’ (289) according to component number. For the total deposited 96 entries in ‘Ambiguous system’, which is a group not included in LLPSDB, 56 of them contain protein(s) with clear information, and the other 40 contain only complex (such as nucleosome).

### 3.2 Improvement in interface of web services

LLPSDB v2.0 allows users to browse, search, submit and download the data, and keeps *Home, Browse, Search, Submit, Statistics, Download* and *Help* modules as LLPSDB. The interactive interface of ‘*Browse*’, ‘*Search*’ and ‘*Download*’ modules are ameliorated for users’ convenience.

**Fig. 1.**
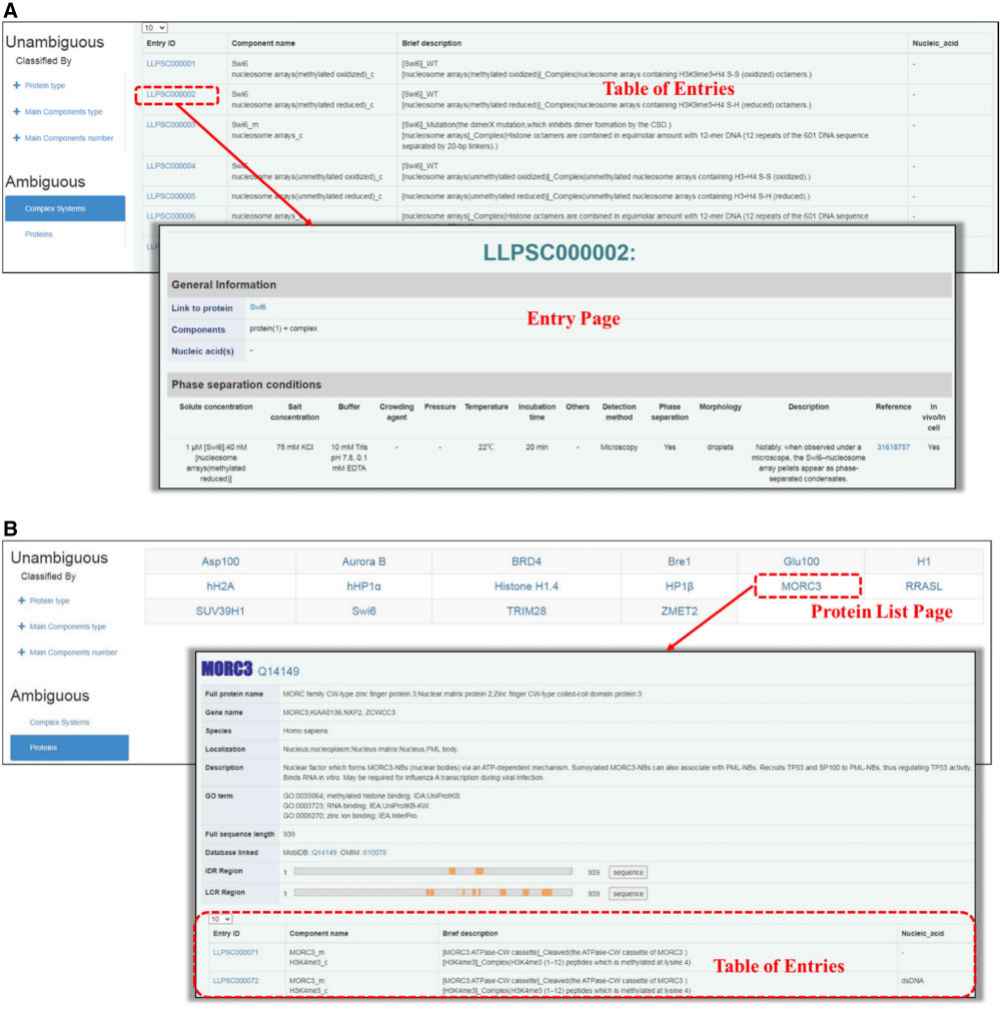
The ‘Ambiguous system’ information designed in *Browse* module of LLPSDB v2.0. (**A**) A brief table (similar to the ‘Table of Entries’ in ‘Unambiguous system’) and a detailed ‘Entry page’ are presented. (**B**) Annotations of specific protein can be browsed through ‘Protein List page’

In *Browse* module, the curated data are grouped into ‘Unambiguous system’ and ‘Ambiguous system’. For ‘Unambiguous system’, same as LLPSDB, three classifications can be browsed based on: protein type, main components type and main components number. The detailed information of independent protein (named as ‘Protein Details page’ in LLPSDB) can be browsed by ‘protein type’ (either ‘natural’ or ‘designed’ protein), through clicking on the name of specific protein on ‘Protein List page’ displaying on the right side. A brief table of entries that this protein involved in (named as ‘Table of Entries’) is presented at the bottom part on ‘Protein Details page’. The ‘Table of Entries’ displays the brief information of main components in each entry, and can be browsed directly for the other two classifications (‘main components type’ and ‘main components number’). More detailed information of each entry (named as ‘Entry page’) is available through clicking on the Entry ID in ‘Table of Entries’, which includes ‘General information’ as well as ‘Phase separation conditions’ of experimental system. For ‘Ambiguous system’, a brief table which includes ‘Component name’, ‘Brief description’ of the system and ‘Nucleic acid’ is presented, and the detailed ‘Entry page’ is accessible through Entry ID (as shown in Fig. 1A), with the recorded ‘General information’ much more reduced. For those specific proteins involved in LLPS with complex, their detailed annotations can be accessed into through ‘Protein List page’ (as shown in Fig. 1B).

**Fig. 2.**
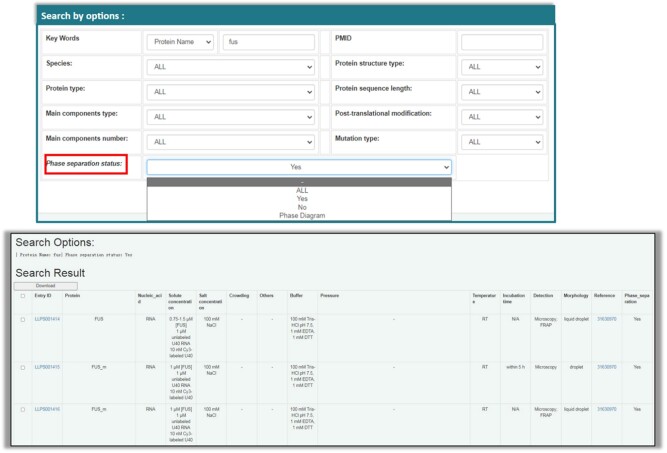
Data in LLPSDB v2.0 can be searched by selecting option ‘Phase separation status’, and an example of part of searched result is presented on the bottom window

In *Search* module, compared with LLPSDB, a new search option ‘phase separation status’ is offered in LLPSDB v2.0, which includes ‘ALL’, ‘Yes’, ‘No’ and ‘Phase Diagram’. A phase diagram in LLPSDB v2.0 was extracted from the screened article and converted into digital table manually to make it more readable. It exhibits the phase separation condition boundary of experimentally investigated system, which can be single component or multi-component. The varying conditions in phase diagram can be temperature, pH, salt concentration, RNA concentration or component concentration, depending on the specific experiment. The added search option provides useful selection such as to screen proteins that can phase separate at physiological conditions. It is convenient for users not only to find the phase behavior of a specific protein quickly, but to build positive or negative datasets for LLPS prediction directly. Users can search the database by ‘phase separation status’ option alone or through combining with other options (Fig. 2). At this time, only data in ‘Unambiguous system’ can be searched. Different from LLPSDB that the searched result is shown in a form as ‘Table of Entries’ (same as in *Browse* module), it is presented in a manner similar as the ‘Phase separation conditions’ part in ‘Entry page’ when the search option ‘phase separation status’ is selected in LLPSDB v2.0. The bottom window of Figure 2 displays an example of partly searched result using the options ‘protein name: Fus’ combined with ‘phase separation status: Yes’. In LLPSDB v2.0, users can download searched result by first ticking the left check boxes of specifically selected lines or the left check box of the first line for all searched result, then clicking on ‘download’ button.

All datasets are free available on the *Download* page. The data for download are grouped into ‘phase separation’, ‘no phase separation’ and ‘phase diagram’ based on ‘phase separation status’ for ‘Unambiguous system’ and ‘Ambiguous system’ respectively. Users can download the classified datasets according to their own needs.

Detailed guidance on the usage of LLPSDB v2.0 can be found on the *Help* page.

## 4 Conclusion

In summary, LLPSDB v2.0 (http://bio-comp.org.cn/llpsdbv2) has been made a significant improvement both in the data quantity and in web service interface compared with previous version LLPSDB. It is more than twice the data volume of LLPSDB. A new class ‘Ambiguous system’ is curated in which part or all components in experimental system are unclear. Totally, LLPSDB v2.0 contained 2917 experimentally validated entries including 586 independent proteins and 6678 corresponding experimental conditions (included 448 phase diagrams). Meanwhile, to provide a more user-friendly interface, we implemented the online service of LLPSDB v2.0 by PHP, improved the *Search* module by adding more options and provided selectable download service. We believe that the updated database provides a more useful resource for better understanding the mechanism of protein LLPS.

To make LLPSDB as an ever-growing comprehensive database, we will continuously update and expand our database by collecting the latest data. Firstly, we may collect and curate more types of liquid-liquid separation systems in future versions, such as those only containing lipids, RNA or DNA and those undergoing LLPS with quantitatively measured information *in vivo*, etc. Furthermore, we are actively using our database to build prediction tools, which may be provided by online service for users in the near future.

## Supplementary Material

btac026_Supplementary_Data
