# Supplementary material for: LLPSDB v2.0: an updated database of proteins undergoing liquid–liquid phase separation in vitro
Source: Bioinformatics. 2022 Jan 13;38(7):2010–4. doi: 10.1093/bioinformatics/btac026 (PMC8963276; doi:10.1093/bioinformatics/btac026)
Supplement: btac026_Supplementary_Data [file btac026_supplementary_data.zip › btac026-suppl_data/SupplementaryMaterials_2022.1.docx]

# Supplementary Table

**Table S1: Species classification of natural proteins in LLPSDB and LLPSDB v2.0**

| **Species Classification** | **Number of protein in LLPSDB** | **Number of proteins in LLPSDB v2.0** |
| --- | --- | --- |
| Eukaryote(Animal) | 149 | 316 |
| Eukaryote(Plant) | 7 | 15 |
| Eukaryote(Fungi) | 24 | 58 |
| Prokaryote | 18 | 30 |
| Virus | 0 | 16 |

**Table S2: GO-tag list of natural proteins in LLPSDB and LLPSDB v2.0**

| **GO-tag** | **Number of proteins in LLPSDB** | **Number of proteins in LLPSDB v2.0** |
| --- | --- | --- |
| RNA binding | 70 | 133 |
| DNA binding | 42 | 118 |
| protein binding | 82 | 194 |
| enzyme binding | 48 | 96 |
| receptor binding | 20 | 29 |
| ion binding | 36 | 94 |
| NTP binding | 23 | 68 |
| chromatin binding | 23 | 46 |
| enzyme activity | 50 | 110 |
| transcription factor activity | 13 | 34 |
| others | 40 | 65 |
| **dimerization activity** | - | 30 |
| **inhibitor activity** | - | 22 |
| **adaptor activity** | - | 27 |
| **activator activity** | - | 47 |
