# Supplementary material for: LLPSDB v2.0: an updated database of proteins undergoing liquid–liquid phase separation in vitro
Source: Bioinformatics. 2022 Jan 13;38(7):2010–4. doi: 10.1093/bioinformatics/btac026 (PMC8963276; doi:10.1093/bioinformatics/btac026)
Supplement: btac026_Supplementary_Data [file btac026_supplementary_data.zip › btac026-suppl_data/SupplementaryMaterials_2022.1.pdf]

Supplementary Table

Table S1: Species classification of natural proteins in LLPSDB and LLPSDB v2.0

Species Classification	Number of protein in LLPSDB	Number of proteins in LLPSDB v2.0
Eukaryote(Animal)	149	316
Eukaryote(Plant)	7	15
Eukaryote(Fungi)	24	58
Prokaryote	18	30
Virus	0	16

Table S2: GO-tag list of natural proteins in LLPSDB and LLPSDB v2.0

GO-tag	Number of proteins in LLPSDB	Number of proteins in LLPSDB v2.0
RNA binding	70	133
DNA binding	42	118
protein binding	82	194
enzyme binding	48	96
receptor binding	20	29
ion binding	36	94
NTP binding	23	68
chromatin binding	23	46
enzyme activity	50	110
transcription factor activity	13	34
others	40	65
**dimerization activity**	-	30
**inhibitor activity**	-	22
**adaptor activity**	-	27
**activator activity**	-	47
